# Inflammatory Arthritis and the Environment: Causes and Consequences of Spondyloarthritis

**DOI:** 10.3390/jpm15060237

**Published:** 2025-06-05

**Authors:** Maurizio Benucci, Edda Russo, Francesca Li Gobbi, Mariangela Manfredi, Maria Infantino

**Affiliations:** 1 Rheumatology Unit, S. Giovanni di Dio Hospital, Azienda USL-Toscana Centro, 50143 Florence, Italy; francesca.ligobbi@uslcentro.toscana.it; 2 Clinical Pathology Laboratory Unit, S. Giuseppe Hospital, Azienda USL-Toscana Centro, 50053 Empoli, Italy; edda.russo@uslcentro.toscana.it; 3 Immunology and Allergology Laboratory Unit, S. Giovanni di Dio Hospital, Azienda USL-Toscana Centro, 50143 Florence, Italy; mariangela.manfredi@uslcentro.toscana.it (M.M.); maria2.infantino@uslcentro.toscana.it (M.I.)

**Keywords:** spondyloarthritis, air pollution, smoke, microbiota, infection, diet

## Abstract

The extensive research and studies conducted over the past decade have greatly improved our comprehension of the pathogenesis and risk factors associated with Spondyloarthritis (SpA). In addition, they have contributed to the advancement of novel therapeutic approaches. Although genetics still represents the primary risk factor for SpA, increasing evidence presented in this review suggests that environmental factors—such as air pollution, smoking, gut microbiota (GM), infections, and diet—also contribute to its pathogenesis. In detail, environmental particulate matters (PMs), which include ligands for the aryl hydrocarbon receptor—a cytosolic transcription factor responsive to toxic substances—facilitate the differentiation of T Helper 17 (Th17) cells, potentially exacerbating the autoinflammatory processes associated with SpA. Furthermore, smoking influences both the cellular and humoral aspects of the immune response, resulting in leukocytosis, impaired leukocyte functionality, and a decrease in various cytokines and soluble receptors, including interleukin (IL) 15, IL-1 receptor antagonist (IL-1Ra), IL-6, soluble IL-6 receptor (sIL-6R), as well as the vascular endothelial growth factor (VEGF) receptor. Studies have indicated that patients with SpA exhibit an increased prevalence of antibodies directed against a conserved epitope shared by the human leukocyte antigen B27 (HLA-B27)- and *Klebsiella nitrogenase*, in comparison to HLA-B27-positive controls. Additionally, current evidence regarding the GM suggests the presence of a gut–joint–skin axis, wherein the disruption of the mucosal barrier by specific bacterial species may enhance permeability to the gut-associated lymphoid tissue (GALT), resulting in localized inflammation mediated by Th1 and Th17 cells, as well as IL-17A. Finally, this review discusses the role of diet in shaping the microbial composition and its contribution to the pathogenesis of SpA. A comprehensive understanding of the mechanisms by which environmental factors influence the pathogenesis and progression of the disease could facilitate the development of novel personalized therapies targeting both external and internal environmental exposures, such as the gut microbial ecosystem.

## 1. Introduction

Spondyloarthritis (SpA) is a chronic immune-mediated disorder characterized by inflammation and structural damage, including the formation of new bone, affecting both the axial and peripheral skeleton. Its global prevalence is approximately 1%, with disease onset typically occurring before the age of 45. Axial SpA (AxSpA) encompasses both radiographic disease, which exhibits structural changes in the sacroiliac joint and is classified according to the modified New York criteria (commonly referred to as ankylosing spondylitis, AS), and non-radiographic disease (nr-axSpA), which does not show such changes on imaging [1].

The human leukocyte antigen B27 (HLA-B27) allele, a major histocompatibility complex (MHC) class I molecule, is recognized as the most significant genetic risk factor for AxSpA. These molecules are expressed on the surface of nucleated cells and are capable of presenting peptides to CD8+ T cells. HLA-B27 is found in 85–90% of AS patients and in 75–90% of those with nr-axSpA, in contrast to its presence in only 5% of the healthy Caucasian population. Beyond HLA-B27, additional MHC class I genes and various non-MHC coding genes have been linked to AxSpA through extensive genome-wide association studies (GWAS) [2]. Among the non-MHC coding genes, those associated with innate immune responses, IL-23/IL-17 signaling pathways, epithelial function, joint and bone remodeling, and antigen presentation have been identified. Some single nucleotide polymorphisms (SNPs) in genes such as ERAP1/2, TNF-α, IL-1A, IL-23A-R, and CTLA-4 appear to be associated with a greater likelihood of developing AS, with a different risk pattern based on ethnicity [3].

A hallmark of SpA is the involvement of enthesis sites, which exhibit microregions characterized by a complete absence of cortical bone. This deficiency allows for direct contact between the underlying trabecular bone and the fibrocartilage of the enthesis *via* microfractures. At these interfaces, the trabecular bone responds to fibrocartilaginous microdamage by initiating a reparative process involving the invasion of blood vessels and mesenchymal connective tissue cells. Evidence of active remodeling is observed in both the thin subentheseal cortical bone and the adjacent trabecular bone, as indicated by the presence of osteoid tissue, osteoclasts, osteoblasts, immune cells, and stem/progenitor cells [4]. The DKK-1 system activates the Wnt/b catenin pathway with action on osteoblasts. Progressive cartilage degradation in inflammatory arthritis not only involves cytokine-mediated catabolic processes but is also strongly influenced by the mechanical and tribological properties of articular cartilage [5].

New evidence suggests that environmental factors, including air pollution, smoking, gut microbiota (GM), infections, and type of diet, play a pathogenetic role in SpA. A gut–joint–skin axis has been suggested, wherein the disruption of the mucosal barrier caused by specific bacterial species may enhance permeability to the gut-associated lymphoid tissue (GALT). This increased permeability can result in localized inflammation that is mediated by Th1 and Th17 cells, as well as in IL-17A.

Inflammation may further support gut dysbiosis and could also trigger systemic diseases. In this context, the modulation of the aforementioned axis could represent a novel personalized approach to mitigate the manifestation of the disease.

## 2. Methods

The scientific literature involving environmental factors and SpA has been reviewed thoroughly. The research was carried out using the PubMed and EMBASE databases, searching the following keywords: spondyloarthritis, air pollution, smoking, gut microbiome, infection, and diet. We selected only English-language articles, excluding abstracts without the main text. In detail, the manuscript has been structured as a narrative review.

## 3. Air Pollution

Outdoor air pollution has the potential to initiate systemic inflammatory responses [6], and chronic exposure to air pollutants has been linked to an elevated risk of developing or worsening certain immune-mediated diseases [7,8]. Specifically, prolonged exposure to particulate matter with a diameter of less than 2.5 μm (PM 2.5) has shown a strong association with poorer outcomes in AS [9]. However, the relationship between other air pollutants, such as sulfur dioxide (SO_2_), ozone (O_3_), nitrogen dioxide (NO_2_), and carbon monoxide (CO), with AS remains uncertain. A case–control study has highlighted a significant correlation between long-term exposure to PM2.5 and adverse outcomes in AS patients [9]. Additionally, air pollution has been shown to exacerbate joint symptoms in patients with AS [6]. However, a cohort study by Park et al. found no association between long-term exposure to environmental PMs and the incidence of SpA [10], suggesting a limited pathogenetic role of PMs in SpA. In addition, air pollution triggers inflammatory immune responses involving innate and adaptive immunity from pulmonary to systemic sites [11,12], thereby contributing to the development and progression of autoimmune diseases [13]. Recent research has highlighted the involvement of TNF and Th17 cells in proinflammatory responses [14,15,16], which play a key role in the immunopathological mechanisms of SpA.

Environmental PMs include ligands for the aryl hydrocarbon receptor, which is a cytosolic transcription factor that reacts to toxic substances. This interaction promotes the differentiation of Th17 cells, potentially exacerbating the autoinflammatory processes associated with SpA [17,18]. However, the relationship between air pollution and the type 3 pulmonary innate lymphoid cell response has not yet been clearly studied [19]. In a nationwide observational study conducted in Taiwan using the Taiwan National Health Insurance Research Database, newly diagnosed SpA patients from 2003 to 2013 were identified and, among them, 584 patients who initiated biologic therapy between 2012 and 2013 were included [20]. The authors investigated the relationship between the initiation of biologic therapy and exposure to air pollutants in the year leading up to the start of treatment. They took care to adjust for potential confounding factors to ensure that their findings accurately reflected the impact of air pollution on the timing of biologic therapy initiation. The initiation of reimbursed biologic therapy demonstrated a positive correlation with CO levels, whereas an inverse association was observed with NO₂ concentrations. However, major limitations of the study included the lack of data on individual smoking status and the potential multicollinearity amongst air pollutants [20].

In general, to mitigate the risk of SpA development associated with air pollution, patients can be advised to limit outdoor exposure, particularly during periods of elevated pollutant concentrations. The use of high-efficiency indoor air purification systems can be recommended to maintain optimal air quality in enclosed environments. When outdoor activity is unavoidable, wearing protective face masks can further reduce the inhalation of harmful airborne particles. Moreover, the continuous monitoring of local air quality indices and adjusting physical activity accordingly can be essential preventative strategies.

Take-home messages:

− Chronic exposure to fine particulate matter (PM 2.5, <2.5 μm in diameter) is strongly associated with worsened clinical outcomes in AS.
− Air pollution provokes inflammatory immune responses involving both innate and adaptive immune mechanisms, with effects extending from the respiratory tract to systemic circulation.
− Recent studies underscore the involvement of TNF and Th17 cells as central drivers of proinflammatory activity, contributing significantly to the immunopathogenesis of AS.

## 4. Smoking Cigarettes and e-Cigarettes

Current smoking, rather than a history of smoking, is identified as a risk factor for SpA and is associated with increased disease activity in those affected. The known proinflammatory and pro-oxidative effects of smoking play a role in triggering the disease in individuals who are genetically predisposed, as well as in advancing nr-axSpA within the broader context of SpA [21]. In the population-based Nord-Trøndelag Health Study, the occurrence of incident AS was found to be correlated with smoking and hypertension [21]. Logistic regression analysis, adjusted for potential confounders, has demonstrated a significant association between current smoking and SpA. Moreover, a meta-analysis evaluating the association between smoking and disease outcomes in Chinese patients with SpA revealed that both current and former smokers exhibited significantly elevated Bath Ankylosing Spondylitis Disease Activity Index (BASDAI) scores and poorer functional capacity compared to nonsmokers [22]. Similarly, among French patients with AxSpA who smoked, greater inflammatory involvement of the sacroiliac joint—detected by magnetic resonance imaging (MRI)—was independently associated with smoking at each visit over a 5-year follow-up period [23]. While there are a lack of data regarding the effects of e-cigarette use in patients with SpA, findings from a murine model of arthritis indicate that nicotine may exacerbate inflammatory arthritis [24]. This implies that nicotine-containing products, including e-cigarettes, may have harmful effects on SpA patients who smoke them. There is also a well-established link between smoking and new bone formation, as observed in SpA and axSpA; however, the underlying mechanisms remain unclear [25,26]. Previous studies suggest that smoking affects both the cellular and humoral components of the immune system, leading to leukocytosis, impaired leukocyte function, and increased levels of certain cytokines as well as soluble receptors such as IL 15, IL-1Ra, IL-6, sIL-6R, and vascular endothelial growth factor receptor-3. Additionally, smoking promotes the generation of reactive oxygen species, triggers tissue hypoxia, and enhances danger signals, similar to the effects of mechanical stress [27,28].

Take-home messages:

− Current smoking, as opposed to a prior history of smoking, appears to be a significant risk factor for AS and is associated with increased disease activity in affected individuals.
− The proinflammatory and pro-oxidative properties of smoking are believed to contribute to disease initiation, particularly in genetically susceptible individuals, and may promote the progression from nr-axSpA to AS.
− While clinical data on e-cigarette use in patients with AS are lacking, findings from a murine model of arthritis indicate that nicotine may exacerbate inflammatory arthritis.

## 5. Intestinal Microbiome

Based on current knowledge of SpA pathogenesis, the human microbiome (the community of microorganisms such as fungi, bacteria, and viruses that reside in the human body) likely plays a significant role in disease evolution, particularly in genetically predisposed individuals. The introduction of commensal bacteria, such as *Bacteroides vulgatus*, into transgenic animal models has been demonstrated to trigger arthritis [29]. Additionally, the transfer of HLA-B27 transgenic rats from a germ-free environment to a conventional rat colony resulted in the onset of SpA symptoms [29]. Moreover, an observational study examining the association between disease activity and infections in Mexican patients with various forms of SpA revealed a higher prevalence of enteric infections among HLA-B27-positive individuals [30]. This result supports the role of both genetics and microbial infections in the development of SpA. In detail, HLA-B27 may contribute to SpA susceptibility by altering the GM composition and presenting a distinct and divergent set of peptides in the intestine. This mechanism establishes a localized environment that facilitates microbial imbalance, immune activation, and excessive IL-23 production, along with other proinflammatory cytokines [31], a phenomenon also observed in HLA-B27-positive healthy individuals [32]. Notably, intestinal barrier dysfunction is more pronounced in SpA patients, their first-degree relatives, and animal models, resulting in increased systemic exposure to potentially harmful gut microorganisms [33]. Moreover, Paneth cells, a distinct subset of secretory epithelial cells within the small intestine, are essential for immune defense and have been identified as a key source of IL-23. This cytokine subsequently stimulates IL-23-responsive immune populations, including group 3 innate lymphoid cells (ILC3), γδ T cells, and mucosal-associated invariant T (MAIT) cells. These activated immune cells migrate from the gut to inflammatory sites central to SpA pathogenesis, such as the entheses [34]. In patients with both SpA and Crohn’s disease (CD), the significant upregulation of IL-23p19 transcripts has been observed in the terminal ileum, suggesting a potential relationship between IL-23 receptor polymorphisms and intestinal inflammation [35]

Several GM families have been implicated in the onset of SpA in humans, including *Bacteroidaceae*, *Porphyromonadaceae*, *Lachnospiraceae*, *Rikenellaceae*, *Prevotellaceae*, and *Ruminococcaceae* [36]. These microbial groups have been correlated with fecal calprotectin levels, an indicator of intestinal inflammation, but not with other clinical disease parameters [37]. A metagenomic analysis of gut microbial DNA from 211 Chinese individuals revealed that SpA patients exhibited increased abundances of *Prevotella copri*, *Prevotella melaninogenica*, and *Prevotella sp.* C561, while showing a decline in *Bacteroides* species [38]. Interestingly, the *Bifidobacterium* genus, commonly used in probiotics, was found to accumulate in SpA patients [38]. Another observational study in Chinese SpA patients confirmed the presence of GM dysbiosis and showed that treatment with TNF inhibitors (TNFi) was associated with the partial restoration of the disrupted microbiome, bringing it closer to that of healthy controls (HCs) [39]. Asymptomatic intestinal inflammation, primarily affecting the terminal ileum, was detected in a substantial proportion of SpA patients (57–70%), with a higher prevalence among those with peripheral arthritis, reinforcing the association between gut dysbiosis and SpA pathogenesis [40]. However, no conclusive evidence has yet established a direct causal link between SpA and subclinical intestinal inflammation [41].

A fecal microbiome observational study involving 103 Chinese SpA patients and 104 healthy controls (HCs) identified distinct alterations in the GM composition. Specifically, *Dorea*, *Megamonas*, and *Blautia* genera were more abundant in SpA patients compared to HCs, whereas *Ruminococcus*, *Lachnospira*, and *Clostridium*_XIVb were reduced. Several pathogenic taxa, including *Micrococcaceae*, *Nocardiaceae*, *Firmicutes*, *Actinomycetales*, *Coprobacter*, *Erysipelotrichaceae*, and *Lactobacillus mucosae*, were associated with disease activity [42]. Ribosomal RNA analyses of intestinal biopsies from 27 ankylosing spondylitis (AS) patients and 15 HCs revealed a strong correlation between intestinal inflammation and specific bacterial species. Notably, increased *Dialister* species expression was linked to higher joint disease activity [43]. Additionally, Breban et al. reported elevated levels of *Ruminococcus gnavus* ribosomal RNA in SpA patients compared to HCs and rheumatoid arthritis (RA) patients [44]. GM dysbiosis has been associated with SpA severity and extra-articular manifestations such as inflammatory bowel disease (IBD), highlighting a shared pathogenic mechanism between the two conditions [45]. In particular, a reduced abundance of *Firmicutes*, notably *Faecalibacterium prausnitzii* and *Clostridium leptum*, alongside increased levels of *Ruminococcus gnavus*, has been observed in both SpA and IBD patients [46]. The GM composition of SpA patients was more divergent from that of HLA-B27-negative HCs than from that of HLA-B27-positive HCs, suggesting a potential influence of HLA-B27 on microbial dysbiosis [32,47,48]. In another observational study, intestinal biopsies from 107 HCs also revealed significant differences between HLA-B27-positive and HLA-B27-negative individuals. HLA-B27-positive subjects exhibited a decline of *Blautia obeum*, *Bacteroides ovatus*, and *Dorea formicigenerans*, along with an increase of *Roseburia species* and the *Neisseriaceae* family [32]. In addition, the levels of *Oscillospiraceae*, *Lachnospiraceae*, and *Bifdobacteriaceae* were also found to differ between HLA-B27-positive and -negative patients [49]. From a pathogenetic perspective, certain bacterial species, such as *Akkermansia muciniphila* and some *Prevotellaceae*, may compromise the intestinal barrier by degrading mucin. This degradation can lead to increased intestinal permeability and disruption of the tight junctions, potentially contributing to disease pathogenesis [50].

Intestinal vascular leakage has also been reported in SpA patients, as evidenced by the translocation of microbial-derived molecules, such as lipopolysaccharide-binding protein, and elevated zonulin levels [51]. Tissue-specific immune responses, particularly at the GALT level, may interact with the GM. Notably, increased IgA levels in SpA have been well documented [52,53], and mucosal antibodies are produced within lymphoid follicles, which are both enlarged and more numerous in the intestines of SpA patients [54]. Additionally, anti-*Saccharomyces cerevisiae* (ASCA), antiflagellin, and *Escherichia coli* antibodies have been proposed as potential biomarkers for SpA, with ASCA levels correlating with disease activity [55,56]. Certain pathogens, such as *Ruminococcus gnavus*, secrete a structurally complex glucomannan polysaccharide capable of stimulating inflammatory cytokine production, including TNFα, via toll-like receptor activation on dendritic cells. This mechanism may explain the link between *Ruminococcus gnavus* and intestinal inflammation in CD [57]. Furthermore, studies have indicated that *Akkermansia* and *Ruminococcus species*—often reduced in AS patients—may be involved in the diminished production of short-chain fatty acids (SCFAs). SCFAs are known to play a protective role by preventing the colonization of pathogenic bacteria and modulating immune responses [58]. Specifically, SCFAs represent a subgroup of fatty acids produced by GM through the fermentation of partially and nondigestible polysaccharides.

Although SpA can be managed with pharmacological treatments, these medications are often associated with significant side effects and complications. On the other side, probiotics, as natural therapeutic agents, have demonstrated efficacy against various manifestations of SpA [59]. They modulate the GM, decreasing susceptibility to SpA-related inflammatory processes. Commercially available probiotics commonly contain species from the genera *Lactobacillus* and *Bifidobacterium*. In a study by Baharav et al. (2004), the administration of either heat-killed or live *Lactobacillus rhamnosus* GG significantly ameliorated experimental arthritis induced by adjuvant or tropomyosin in rats [60]. The therapeutic effect of *L. rhamnosus* GG is believed to be mediated through the inhibition of the p38 mitogen-activated protein kinase (p38 MAPK) signaling pathway, leading to the reduced expression of cyclooxygenase-2 (COX-2) [61]. Moreover, VSL#3, a commercially available multi-strain probiotic formulation, contains eight bacterial strains. The administration of VSL#3 to IL-10-deficient mice was associated with the reduced production of TNF-α and IFN-α in the gut mucosa—both cytokines were implicated in the SpA pathogenesis [62]. Therefore, the use of probiotics as an alternative or adjunctive therapeutic approach warrants consideration, potentially offering a safer and more sustainable strategy for the management or attenuation of SpA progression.

In summary, current evidence supports the concept of a gut–joint–skin axis, wherein the disruption of the mucosal barrier by specific bacterial species enhances permeability to the GALT, triggering localized inflammation driven by Th1 and Th17 cells and IL-17A. Given the well-established associations with HLA-B27, genetic factors likely contribute to shaping the microbiota and influencing both local and systemic immune responses, thereby facilitating antigen presentation and amplifying immune reactivity to intestinal dysbiosis [63].

Take-home messages:

− The introduction of commensal bacteria, such as *Bacteroides vulgatus*, into transgenic animal models has been shown to induce the development of arthritis.
− HLA-B27 may confer susceptibility to AS by altering the composition of the GM and presenting a distinct repertoire of peptides within the intestinal environment, thereby promoting microbial dysbiosis, local inflammation, and the enhanced production of IL-23 and other proinflammatory mediators.
− Paneth cells, a specialized subset of antimicrobial epithelial cells in the small intestine, secrete IL-23 and activate IL-23-responsive immune populations—such as ILC3s, γδ T cells, and MAIT cells—which can migrate from the gut to peripheral inflammatory sites, including the entheses, contributing to the pathogenesis of SpA.

### 5.1. Infections

*Klebsiella pneumoniae* has been implicated in the pathogenesis of SpA, as an increased fecal load of this bacterium has been observed in patients with active disease [64]. Additionally, another study found that HLA-B27-positive individuals exhibited a reduced lymphocyte response to *Klebsiella* antigens [65]. However, these initial findings have not been consistently replicated in subsequent research [66,67]. The hypothesis of molecular mimicry between *Klebsiella* capsular antigens (as well as those of other *Enterobacteriaceae*) and HLA-B27 has been proposed, supported by evidence of cross-reactivity between antigens from various Gram-negative bacteria and lymphocytes from HLA-B27-positive individuals [68]. It was also reported that patients with SpA had a higher frequency of antibodies to a homologous region shared by HLA-B27 and *Klebsiella nitroase* compared to HLA-B27-positive controls [69]. This suggests that AS may represent an HLA-B27-directed autoimmune response initially triggered by *Klebsiella pneumoniae* nitrate proteins. However, this observation has not been independently replicated [70]. Alternative studies have suggested that active SpA is instead characterized by increased IgA antibody levels against various *Enterobacteriaceae*, irrespective of HLA-B27 status, thereby questioning the molecular mimicry hypothesis [71,72]. Another proposed mechanism involves the modification of specific MHC-associated gene products by a soluble cell wall factor originating from the *Klebsiella* K43 plasmid, potentially contributing to the development of HLA-B27-related arthropathy [73]. Subsequent investigations have failed to confirm this theory [74,75]. Research on SpA patients and their relatives, regardless of whether they have a family history of SpA, has not identified a distinct antibody response to *Klebsiella pneumoniae* in association with SpA [76]. A recent systematic review [77] referenced one study indicating that immune responses to *Klebsiella* proteins persist in SpA patients [78], while another discussed the rising interest in “low-starch” dietary approaches and “anti-*Klebsiella*” supplements [79], though current evidence does not strongly support their effects on disease progression. Furthermore, earlier findings linking *Klebsiella* to GM imbalances have not been corroborated by more recent microbiome analyses [80].

### 5.2. Other Infections

A study of 61,550 individuals demonstrated a significantly increased risk of developing SpA with an adjusted hazard ratio (aHR) of 1.77 (95% CI = 1.26–2.53). This association was observed after six years of follow-up in individuals with *Candida* exposure at baseline [81]. Human *Papilloma virus* (HPV) infection was associated with a 1348-fold greater risk of AS compared to the non-HPV cohort, based on data from a total population of 66,314 patients with an HPV infection [82]. Among the autoimmune diseases in individuals with human immunodeficiency virus (HIV), AS showed the strongest association, with an adjusted hazard ratio of 1.82, based on an analysis of 4245 HIV-positive patients [83]. Finally, in one study, David et al. evaluated the fecal microbiome of IBD patients with or without peripheral SpA. Coupling IgA-coated microbiota sorting with 16S ribosomal RNA-based analysis (IgA-seq) revealed a selective enrichment in IgA-coated *Escherichia coli* in patients with CD-associated SpA (CD-SpA) compared to those with CD alone [84].

## 6. Diet

Diet plays a crucial role in shaping the composition and activity of the GM by modulating its gene expression and metabolite production. Zhang et al. [42] proposed that alterations in the GM observed in SpA are also influenced by dietary factors. Additionally, Lawrence et al. [85] demonstrated that short-term modifications in macronutrient intake could restructure the GM and decrease interindividual variability in microbial gene expression. This finding suggests that the GM can rapidly adapt to dietary changes, potentially influencing the nutritional management of patients with AS. Since diet is an easily modifiable environmental factor, it has been proposed that a dietary intervention could serve as a potential strategy for the prevention or treatment of the disease [86]. In addition, AS patients themselves consider diet more important than medications [87]. Recent research indicates that a diet high in starch and fat, commonly associated with the “Western diet”, may contribute to the development of autoimmune disorders by compromising the integrity of the intestinal barrier and altering the composition and metabolic functions of the GM [88]. Certain pathogenic gut bacteria thrive on dietary starch; thus, reducing the intake of starch-rich foods such as bread, sweets, and potatoes could be advantageous for SpA patients. Studies have shown that adherence to a low-starch diet results in a reduction in total serum IgA levels in both healthy individuals and patients with SpA, potentially contributing to decreased inflammation and symptomatic improvement in individuals with AS [87].

Similarly, low-fructose diets have been linked to reductions in inflammatory markers, oxidative stress, and metabolic syndrome [89]. Conversely, insufficient fiber consumption has been associated with a higher prevalence of SpA [90]. Dietary fiber undergoes fermentation in the colon, which supports microbial diversity, strengthens the intestinal barrier, and stimulates the production of SCFAs, all of which contribute to maintaining GM balance and preventing inflammation [91,92,93]. In particular, fiber-rich diets promote the synthesis of SCFAs such as acetate and butyrate. The immune tolerance to dietary antigens relies on CD103⁺ dendritic cells (DCs) in the gut mucosa, which facilitate the differentiation of regulatory T (Treg) cells. SCFAs enhance intestinal immune tolerance by increasing retinal dehydrogenase activity in CD103⁺ DCs, a process dependent on dietary vitamin A. This immunoprotective mechanism supports IgA production and strengthens mucosal germinal center responses as well as T follicular helper cell activity. In contrast, mice lacking SCFA receptors GPR43 or GPR109A exhibit heightened food allergy responses and a diminished population of CD103⁺ DCs. Therefore, adequate fiber and vitamin A intake is essential to regulate immune responses to dietary antigens and safeguard gastrointestinal health [94]. Additionally, the consumption of low-fat milk has been associated with increased GM diversity, whereas high-fat dairy intake correlates with reduced microbial diversity [95]. The Mediterranean diet, which emphasizes fiber-rich whole grains, vegetables, and unsaturated fats such as olive oil and nuts, shares similarities with anti-inflammatory dietary patterns [96]. Adherence to this diet has been linked to decreased levels of inflammatory biomarkers [97,98], suggesting its potential as a beneficial strategy for SpA management.

In addition, eicosapentaenoic acid (EPA) and docosahexaenoic acid (DHA), omega-3 polyunsaturated fatty acids, play a modulatory role in inflammation and immune function, both central to the pathogenesis of SpA. Through the generation of pro-resolving lipid mediators such as resolvins and protectins, EPA and DHA promote the resolution of inflammation. They also reduce proinflammatory cytokine levels (e.g., TNF-α, IL-6), regulate immune cell activity—particularly Th17 cells—and may protect joint and bone structures by inhibiting matrix-degrading enzymes [99]. While these mechanisms suggest potential therapeutic value in SpA, further clinical research is required to confirm their efficacy.

Moreover, α-Gal, a food-derived allergen and the primary natural antigen found in mammalian red meat, has been implicated in SpA pathogenesis [100]. Consequently, allergenic foods such as beef, pork, and crab may need to be eliminated from the diet of SpA patients to reduce potential immune activation.

Take-home messages:

− Zhang et al. proposed that alterations in the GM observed in AS may have been influenced by dietary factors.
− Recent research indicates that the Western diet—characterized by a high intake of starch and saturated fats—may increase the risk of autoimmune diseases by impairing gut barrier integrity and altering microbial composition and metabolism.
− A low-starch diet has been shown to reduce total serum IgA levels in both HCs and patients with AS, potentially leading to decreased inflammation and symptom relief.
− The Mediterranean diet, rich in dietary fiber, whole grains, vegetables, and unsaturated fats such as olive oil and nuts, shares features with anti-inflammatory dietary patterns and may offer benefits in managing inflammatory conditions like AS.

## 7. Genetic and Environmental Factors

Multiple studies have identified HLA-B27 as the most significant genetic marker associated with the development of SpA. There are two broad theoretical hypotheses regarding its pathogenic role: (1) HLA-B27 presents antigenic peptides to cytotoxic T cells to guide the immune response; (2) HLA-B27 induces ER stress and autophagic responses to promote inflammation.

A GWAS study has identified the enzyme encoding endoplasmic reticulum aminopeptidase 1 (ERAP1) as a research risk factor for SpA. The ERAP1 polymorphism leads to endoplasmic reticulum (ER) stress, a condition that may also arise from HLA-B27 misfolding. Both HLA-B27 and ERAP1 are thought to contribute to the activation of the IL-23/IL-17 signaling pathway, thereby promoting inflammation and immune responses [101]. Animal models displaying arthritic and colitis phenotypes, particularly HLA-B27 transgenic rats, have been crucial to understanding the role of GM in SpA. These models have demonstrated that GM is essential for the development of SpA-like symptoms. For example, when HLA-B27 transgenic rats are raised in a germ-free environment, they do not develop SpA [102]. However, when these rats are selectively recolonized with anaerobic bacteria such as *Bacteroides spp.* (and particularly *B. vulgatus*), they develop colitis and arthritis, closely mimicking human SpA [27].

Thus, the elevated prevalence of subclinical intestinal inflammation in patients with spondyloarthritis (approximately 60% of cases), along with increasing evidence of gut dysbiosis in these individuals, reinforces the notion that the GM plays a role in the manifestations of SpA. In addition, assuming that SpA is a disease driven by a type 3 immune response, most of the IL-17 producing cells are represented by mucosal-derived cells such as the ILC3s, MAIT cells, γδ T cells, and tissue-resident memory T (TRM) cells. This evidence, together with the demonstration of the intestinal origin of the inflammatory cells found in the peripheral blood, synovial fluid, and inflamed marrow of patients with SpA, supports the hypothesis of the existence of a gut–joint–enthesis axis in SpA [103]. It is therefore possible to speculate that the modulation of the abovementioned axis, which can target both the microbial and inflammatory aspects of the disease, may represent a new therapeutic and personalized approach to reduce the manifestations of SpA.

## 8. Conclusions

Extensive research over the past decade has greatly enhanced our understanding of the pathogenesis and risk factors of SpA, facilitating the development of innovative therapeutic approaches. While genetics remains the predominant risk factor for SpA, various environmental triggering factors have also been implicated in its onset, either independently or in combination with genetic predisposition. Smoking—including the use of e-cigarettes—should be actively discouraged in individuals at elevated risk for SpA, particularly those with a family history of the disease or who are HLA-B27-positive. Among the environmental factors, substantial evidence strongly supports the role of the GM in SpA pathogenesis, although certain aspects of its involvement remain unclear. Future research should prioritize defining the specific microbial species associated with AS and clarifying the mechanisms through which the GM contributes to disease onset and progression. Striking a balance between mitigating known risk factors and developing disease-modifying therapies that target key pathogenic pathways will be crucial in reducing both the prevalence and severity of SpA. Table 1 provides a summary of how environmental factors may interact with genetic predispositions, soluble mediators, and cellular components of the immune system to initiate and drive the pathogenic mechanisms underlying SpA.

## Figures and Tables

**Table 1:** Environmental factors and spondyloarthritis.

Environmental Factors	Pathogenic Link	Pathogenesis	References
Particulate matter <2,5M SO_2_-O_3_-NO_2_	Aryl hydrocarbon receptor	Lymphocytes Th-17-TNF	[9,10,11,17,18]
Nicotine-containing products, including e-cigarettes	Soluble receptors	IL 15, IL-1Ra, IL-6, sIL- 6R and vascular endothelial growth factor receptor-3	[2,25,26,28]
*Bacteroidaceae*, *Porphyromonadaceae*, *Lachnospiraceae*, *Rikenellaceae*, *Prevotellaceae*	Paneth cells	IL-23-responsive immune populations, including group 3 innate lymphoid cells (ILC3) and γδ T cells	[36,37,38]
*Klebsiella*, *Enterobacteriaceae*	HLA-B27	Immunoglobulin A (IgA)-coated microbiota	[65,71,72,84]
Diet high in starch and fat, Western diet	CD103⁺ dendritic cells (DCs)	T follicular helper cell activity	[85,91,94]

## References

[1] Sieper J., Poddubnyy D. (2017). Axial spondyloarthritis. Lancet.

[2] Brown M.A., Kenna T., Wordsworth B.P. (2016). Genetics of ankylosing spondylitis—Insights into pathogenesis. Nat. Rev. Rheumatol..

[3] Fattorini F., Gentileschi S., Cigolini C., Terenzi R., Pata A.P., Esti L., Carli L. (2023). Axial spondyloarthritis: One year in review 2023. Clin. Exp. Rheumatol..

[4] De Cata A., Inglese M., Rubino R., Molinaro F., Mazzoccoli G. (2016). The synovio-entheseal complex in enthesoarthritis. Clin. Exp. Med..

[5] Krakowski P., Rejniak A., Sobczyk J., Karpiński R. (2024). Cartilage Integrity: A Review of Me-chanical and Frictional Properties and Repair Approaches in Osteoarthritis. Healthcare.

[6] Hiraiwa K., van Eeden S.F. (2013). Contribution of lung macrophages to the infammatory responses induced by exposure to air pollutants. Mediat. Infamm..

[7] Adami G., Pontalti M., Cattani G., Rossini M., Viapiana O., Orsolini G., Benini C., Bertoldo E., Fracassi E., Gatti D. (2022). Association between long-term exposure to air pollution and immunemediated diseases: A population-based cohort study. RMD Open.

[8] Ziadé N., Bouzamel M., Mrad-Nakhlé M., Karam G.A., Hmamouchi I., Abouqal R., Farah W. (2021). Prospective correlational time-series analysis of the influence of weather and air pollution on joint pain in chronic rheumatic diseases. Clin. Rheumatol..

[9] Soleimanifar N., Nicknam M.H., Bidad K., Jamshidi A.R., Mahmoudi M., Mostafaei S., Hosseini-Khah Z., Nikbin B. (2019). Effect of food intake and ambient air pollution exposure on ankylosing spondylitis disease activity. Adv. Rheumatol..

[10] Park J.S., Choi S., Kim K., Chang J., Kim S.M., Kim S.R., Lee G., Son J.S., Kim K.H., Lee E.Y. (2021). Association of particulate matter with autoimmune rheumatic diseases among adults in South Korea. Rheumatology.

[11] Zhao C.-N., Xu Z., Wu G.-C., Mao Y.-M., Liu L.-N., Wu Q., Dan Y.-L., Tao S.-S., Zhang Q., Sam N.B. (2019). Emerging role of air pollution in autoimmune diseases. Autoimmun. Rev..

[12] Glencross D.A., Ho T.R., Camina N., Hawrylowicz C.M., Pfeffer P.E. (2020). Air pollution and its efects on the immune system. Free Radic. Biol. Med..

[13] Gawda A., Majka G., Nowak B., Marcinkiewicz J. (2017). Air pollution, oxidative stress, and exacerbation of autoimmune diseases. Cent. Eur. J. Immunol..

[14] Cox F.A., Stiller-Winkler R., Hadnagy W., Ranft U., Idel H. (1999). Soluble tumor necrosis factor receptor (sTNF RII) in sera of children and traffic-derived particulate air pollution. Zentralbl. Hyg. Umweltmed..

[15] Nakamura R., Inoue K.I., Fujitani Y., Kiyono M., Hirano S., Takano H. (2012). Effects of nanoparticle-rich diesel exhaust particles on IL-17 production in vitro. J. Immunotoxicol..

[16] Mann E.H., Ho T.R., Pfeffer P.E., Matthews N.C., Chevretton E., Mudway I., Kelly F.J., Hawrylowicz C.M. (2017). Vitamin D counteracts an IL-23-dependent IL-17A(+) IFN-gamma(+) response driven by urban particulate matter. Am. J. Respir. Cell Mol. Biol..

[17] Veldhoen M., Hirota K., Westendorf A.M., Buer J., Dumoutier L., Renauld J.C., Stockinger B. (2008). The aryl hydrocarbon receptor links TH17-cell-mediated autoimmunity to environmental toxins. Nature.

[18] van Voorhis M., Knopp S., Julliard W., Fechner J.H., Zhang X., Schauer J.J., Mezrich J.D. (2013). Exposure to atmospheric particulate matter enhances Th17 polarization through the aryl hydrocarbon receptor. PLoS ONE.

[19] Estrella B., Naumova E.N., Cepeda M., Voortman T., Katsikis P.D., Drexhage H.A. (2019). Effects of air pollution on lung innate lymphoid cells: Review of in vitro and in vivo experimental studies. Int. J. Environ. Res. Public Health.

[20] Kao C.-M., Huang W.-N., Chen Y.-H., Chen H.-H. (2023). Association between air pollutants and initiation of biological therapy in patients with ankylosing spondylitis: A nationwide, population-based, nested case–control study. Arthritis Res. Ther..

[21] Videm V., Cortes A., Thomas R., Brown M.A. (2014). Current smoking is associated with incident ankylosing spondylitis—The HUNT population-based Norwegian health study. J. Rheumatol..

[22] Zhang H., Wan W., Liu J., Dai S., Zou Y., Qian Q., Ding Y., Xu X., Ji H., He H. (2018). Smoking quantity determines disease activity and function in Chinese patients with ankylosing spondylitis. Clin. Rheumatol..

[23] Nikiphorou E., Ramiro S., Sepriano A., Ruyssen-Witrand A., Landewé R.B., van der Heijde D. (2020). Do smoking and socioeconomic factors influence Imaging Outcomes in Axial Spondyloarthritis? Five-Year Data From the DESIR Cohort. Arthritis Rheumatol..

[24] Lee J., Luria A., Rhodes C., Raghu H., Lingampalli N., Sharpe O., Rada B., Sohn D.H., Robinson W.H., Sokolove J. (2017). Nicotine drives neutrophil extracellular traps formation and accelerates collagen-induced arthritis. Rheumatology.

[25] Poddubnyy D., Haibel H., Listing J., Märker-Hermann E., Zeidler H., Braun J., Sieper J., Rudwaleit M. (2012). Baseline radiographic damage, elevated acute-phase reactant levels, and cigarette smoking status predict spinal radiographic progression in early axial spondylarthritis. Arthritis Rheum..

[26] Ward M.M., Hendrey M.R., Malley J.D., Learch T.J., Davis J.C., Reveille J.D., Weisman M.H. (2009). Clinical and immunogenetic prognostic factors for radiographic severity in ankylosing spondylitis. Arthritis Care Res..

[27] Sopori M. (2002). Effects of cigarette smoke on the immune system. Nat. Rev. Immunol..

[28] Pacheco-Tena C., González-Chávez S.A. (2015). The danger model approach to the pathogenesis of the rheumatic diseases. J. Immonol. Res..

[29] Rath H.C., Herfarth H.H., Ikeda J.S., Grenther W.B., Hamm T.E., Balish E., Taurog J.D., Hammer R.E., Wilson K.H., Sartor R.B. (1996). Normal luminal bacteria, especially Bacteroides species, mediate chronic colitis, gastritis, and arthritis in HLA-B27/human beta2 microglobulin transgenic rats. J. Clin. Investig..

[30] Martínez A., Pacheco-Tena C., Vázquez-Mellado J., Burgos-Vargas R. (2004). Relationship between disease activity and infection in patients with spondyloarthropathies. Ann. Rheum. Dis..

[31] Rosenbaum J.T., Davey M.P. (2011). Time for a gut check: Evidence for the hypothesis that HLA–B27 predisposes to ankylosing spondylitis by altering the microbiome. Arthritis Rheum..

[32] Asquith M., Sternes P.R., Costello M.E., Karstens L., Diamond S., Martin T.M., Li Z., Marshall M.S., Spector T.D., le Cao K.A. (2019). HLA Alleles Associated With Risk of Ankylosing Spondylitis and Rheumatoid Arthritis Influence the Gut Microbiome. Arthritis Rheumatol..

[33] Martínez-González O., Cantero-Hinojosa J., Paule-Sastre P., Gómez-Magán J.C., Salvatierra-Ríos D. (1994). Intestinal permeability in patients with ankylosing spondylitis and their healthy relatives. Br. J. Rheumatol..

[34] Sharif K., Bridgewood C., Dubash S., McGonagle D. (2020). Intestinal and enthesis innate immunity in early axial spondyloarthropathy. Rheumatology.

[35] Ciccia F., Bombardieri M., Principato A., Giardina A., Tripodo C., Porcasi R., Peralta S., Franco V., Giardina E., Craxi A. (2009). Overexpression of interleukin-23, but not interleukin-17, as an immunologic signature of subclinical intestinal inflammation in ankylosing spondylitis. Arthritis Rheum..

[36] Costello M.E., Ciccia F., Willner D., Warrington N., Robinson P.C., Gardiner B., Marshall M., Kenna T.J., Triolo G., Brown M.A. (2015). Brief report: Intestinal dysbiosis in ankylosing spondylitis. Arthritis Rheumatol..

[37] Klingberg E., Magnusson M.K., Strid H., Deminger A., Ståhl A., Sundin J., Simrén M., Carlsten H., Öhman L., Forsblad-D’elia H. (2019). A distinct gut microbiota composition in patients with ankylosing spondylitis is associated with increased levels of fecal calprotectin. Arthritis Res. Ther..

[38] Wen C., Zheng Z., Shao T., Liu L., Xie Z., Le Chatelier E., He Z., Zhong W., Fan Y., Zhang L. (2017). Quantitative metagenomics reveals unique gut microbiome biomarkers in ankylosing spondylitis. Genome Biol..

[39] Yin J., Stemes P.R., Wang M., Song J., Morrison M., Li T., Zhou L., Wu X., He F., Zhu J. (2020). Shotgun metagenomics reveals an enrichment of potentially crossreactive bacterial epitopes in ankylosing spondylitis patients, as well as the effects of TNFi therapy upon microbiome composition. Ann. Rheum. Dis..

[40] Mielants H., Veys E.M., Cuvelier C., de Vos M. (1988). Ileocolonoscopic findings in seronegative spondylarthropathies. Br. J. Rheumatol..

[41] Klingberg E., Oleröd G., Hammarsten O., Forsblad-D’elia H. (2016). The vitamin D status in ankylosing spondylitis in relation to intestinal inflammation, disease activity, and bone health: A cross-sectional study. Osteoporos. Int..

[42] Zhang L., Han R., Zhang X., Fang G., Chen J., Li J., Xu S., Qian L., Chen W., Pan F. (2019). Fecal microbiota in patients with ankylosing spondylitis: Correlation with dietary factors and disease activity. Clin. Chim. Acta.

[43] Tito R.Y., Cypers H., Joossens M., Varkas G., Van Praet L., Glorieus E., Van den Bosch F., De Vos M., Raes J., Elewaut D. (2017). Brief report: Dialister as a microbial marker of disease activity in spondyloarthritis. Arthritis Rheumatol..

[44] Breban M., Tap J., Leboime A., Said-Nahal R., Langella P., Chiocchia G., Furet J.P., Sokol H. (2017). Faecal microbiota study reveals specifc dysbiosis in spondyloarthritis. Ann. Rheum. Dis..

[45] Mielants H., De Keyser F., Baeten D., Van den Bosch F. (2005). Gut inflammation in the spondyloarthropathies. Curr. Rheumatol. Rep..

[46] Gill T., Asquith M., Rosenbaum J.T., Colbert R.A. (2015). The intestinal microbiome in spondyloarthritis. Curr. Opin. Rheumatol..

[47] Berland M., Meslier V., Berreira Ibraim S., Le Chatelier E., Pons N., Maziers N., Thirion F., Gauthier F., Plaza Oñate F., Furet J.P. (2023). Both disease activity and HLA-B27 status are associated with gut microbiome dysbiosis in spondyloarthritis patients. Arthritis Rheumatol..

[48] Stoll M.L., Sawhney H., Wells P.M., Sternes P.R., Reveille J.D., Morrow C.D., Steves C.J., Brown M.A., Gensler L.S. (2023). The faecal microbiota is distinct in HLA-B27+ ankylosing spondylitis patients versus HLA-B27+ healthy controls. Clin. Exp. Rheumatol..

[49] Vallier M., Segurens B., Larsonneur E., Meyer V., Ferreira S., Caloustian C., Deleuze J.F., Dougados M., Chamaillard M., Miceli-Richard C. (2023). Characterisation of gut microbiota composition in patients with axial spondyloarthritis and its modulation by TNF inhibitor treatment. RMD Open.

[50] Mo C., Lou X., Xue J., Shi Z., Zhao Y., Wang F., Chen G. (2024). The influence of Akkermansia muciniphila on intestinal barrier function. Gut. Pathog..

[51] Ciccia F., Guggino G., Rizzo A., Alessandro R., Luchetti M.M., Milling S., Saieva L., Cypers H., Stampone T., Di Benedetto P. (2017). Dysbiosis and zonulin upregulation alter gut epithelial and vascular barriers in patients with ankylosing spondylitis. Ann. Rheum. Dis..

[52] Veys E., van Leare M. (1973). Serum IgG, IgM, and IgA levels in ankylosing spondylitis. Ann. Rheum. Dis..

[53] Laurent M.R., Panayi G.S. (1983). Acute-phase proteins and serum immunoglobulins in ankylosing spondylitis. Ann. Rheum. Dis..

[54] Demetter P., Van Huysse J.A., De Keyser F., Van Damme N., Verbruggen G., Mielants H., De Vos M., Veys E.M., Cuvelier C.A. (2002). Increase in lymphoid follicles and leukocyte adhesion molecules emphasizes a role for the gut in spondyloarthropathy pathogenesis. J. Pathol..

[55] Romero-Sánchez C., Bautista-Molano W., Parra V., De Avila J., Rueda J.C., Bello-Gualtero J.M., Londoño J., Valle-Oñate R. (2017). Gastrointestinal symptoms and elevated levels of anti-saccharomyces cerevisiae antibodies are associated with higher disease activity in Colombian patients with spondyloarthritis. Int. J. Rheumatol..

[56] Wallis D., Asaduzzaman A., Weisman M., Haroon N., Anton A., McGovern D., Targan S., Inman R. (2013). Elevated serum anti-fagellin antibodies implicate subclinical bowel infammation in ankylosing spondylitis: An observational study. Arthritis Res. Ther..

[57] Henke M.T., Kenny D.J., Cassilly C.D., Vlamakis H., Xavier R.J., Clardy J. (2019). Ruminococcus gnavus, a member of the human gut microbiome associated with Crohn’s disease, produces an infammatory polysaccharide. Proc. Natl. Acad. Sci. USA.

[58] Smith P.M., Howitt M.R., Panikov N., Michaud M., Gallini C.A., Bohlooly-y M., Glickman J.N., Garrett W.S. (2013). The microbial metabolites, short-chain fatty acids, regulate colonic Treg cell homeostasis. Science.

[59] Singh N., Yadav H., Marotta F., Singh V. (2017). Probiotics—A Probable Therapeutic Agent For Spondyloarthropathy. Int. J. Probiotics Prebiotics..

[60] Baharav E., Weinberger A., Mor F., Halpern M. (2004). Lactobacillus GG bacteria ameliorate arthritis in Lewis rats. J. Nutr..

[61] Yan F., Polk D.B. (2002). Probiotic bacterium prevents cytokine-induced apoptosis in intestinal epithelial cells. J. Biol. Chem..

[62] Asquith M., Elewaut D., Lin P., Rosenbaum J.T. (2014). The role of the gut and microbes in the pathogenesis of spondyloarthritis. Best Pract. Res. Clin. Rheumatol..

[63] Song Z.-Y., Yuan D., Zhang S.-X. (2022). Role of the microbiome and its metabolites in ankylosing spondylitis. Front. Immunol..

[64] Ebringer R., Cooke D., Cawdell D.R., Cowling P., Ebringer A. (1977). Ankylosing spondylitis: Klebsiella and HL-A B27. Rheumatol. Rehabil..

[65] Seager K., Bashir H.V., Geczy A.F., Edmonds J., de Vere-Tyndall A. (1979). Evidence for a specific B27-associated cell surface marker on lymphocytes of patients with ankylosing spondylitis. Nature.

[66] Warren R.E., Brewerton D.A. (1980). Faecal carriage of klebsiella by patients with ankylosing spondylitis and rheumatoid arthritis. Ann. Rheum. Dis..

[67] Stebbings S., Munro K., Simon M.A., Tannock G., Highton J., Harmsen H., Welling G., Seksik P., Dore J., Grame G. (2002). Comparison of the faecal microflora of patients with ankylosing spondylitis and controls using molecular methods of analysis. Rheumatology.

[68] Ebringer A. (1983). The cross-tolerance hypothesis, HLA-B27 and ankylosing spondylitis. Br. J. Rheumatol..

[69] Schwimmbeck P.L., Yu D.T., Oldstone M.B. (1987). Autoantibodies to HLA B27 in the sera of HLA B27 patients with ankylosing spondylitis and Reiter’s syndrome. Molecular mimicry with Klebsiella pneumoniae as potential mechanism of autoimmune disease. J. Exp. Med..

[70] de Vries D.D., Dekker-Saeys A.J., Gyodi E., Bohm U., Ivanyi P. (1992). Absence of autoantibodies to peptides shared by HLA-B27.5 and Klebsiella pneumoniae nitrogenase in serum samples from HLA-B27 positive patients with ankylosing spondylitis and Reiter’s syndrome. Ann. Rheum. Dis..

[71] Mäki-Ikola O., Lehtinen K., Nissiläa M., Granfors K. (1994). IgM, IgA and IgG class serum antibodies against Klebsiella pneumoniae and Escherichia coli lipopolysaccharides in patients with ankylosing spondylitis. Br. J. Rheumatol..

[72] Kijlstra A., Luyendijk L., van der Gaag R., van Kregten E., Linssen A., Willers J.M. (1986). IgG and IgA immune response against klebsiella in HLA-B27-associated anterior uveitis. Br. J. Ophthalmol..

[73] Geczy A.F., Alexander K., Bashir H.V., Edmonds J.P. (1980). Characterization of a factor(s) present in Klebsiella culture filtrates that specifically modifies an HLA-B27-associated cell-surface component. J. Exp. Med..

[74] Trapani J.A., McKenzie I.F. (1985). Klebsiella ‘modifying factor’: Binding studies with HLA-B27+ and B27− lymphocytes. Ann. Rheum. Dis..

[75] Ngo K.Y., Rochu D., D’Ambrosio A.M., Muller J.Y., Lucotte G. (1984). Klebsiella plasmid K21 is not involved in the aetiology of ankylosing spondylitis. Exp. Clin. Immunogenet..

[76] Sprenkels S.H.D., Van Kregten E., Feltkamp T.E.W. (1996). IgA antibodies against Klebsiella and other Gram-negative bacteria in ankylosing spondylitis and acute anterior uveitis. Clin. Rheumatol..

[77] Zhang L., Zhang Y.-J., Chen J., Huang X.-L., Fang G.-S., Yang L.-J., Duan Y., Wang J. (2018). The association of HLA-B27 and Klebsiella pneumoniae in ankylosing spondylitis: A systematic review. Microb. Pathog..

[78] Puccetti A., Dolcino M., Tinazzi E., Moretta F., D’angelo S., Olivieri I., Lunardi C. (2017). Antibodies directed against a peptide epitope of a klebsiella pneumoniae-derived protein are present in ankylosing spondylitis. PLoS ONE.

[79] Rashid T., Wilson C., Ebringer A. (2015). Raised incidence of ankylosing spondylitis among Inuit populations could be due to high HLA-B27 association and starch consumption. Rheumatol. Int..

[80] Breban M., Beaufrère M., Glatigny S. (2019). The microbiome in spondyloarthritis. Best Pract. Res. Clin. Rheumatol..

[81] Wei J.C.-C., Chou M.-C., Huang J.-Y., Chang R., Hung Y.-M. (2020). The association between *Candida* infection and ankylosing spondylitis: A population-based matched cohort study. Curr. Med. Res. Opin..

[82] Wei C.-Y., Lin J.-Y., Wang Y.-T., Huang J.-Y., Wei J.C.-C., Chiou J.-Y. (2020). Risk of ankylosing spondylitis following human papillomavirus infection: A nationwide, population-based, cohort study. J. Autoimmun..

[83] Damba J.J., Laskine M., Jin Y., Sinyavskaya L., Durand M. (2021). Incidence of autoimmune diseases in people living with HIV compared to a matched population: A cohort study. Clin. Rheumatol..

[84] Viladomiu M., Kivolowitz C., Abdulhamid A., Dogan B., Victorio D., Castellanos J.G., Woo V., Teng F., Tran N.L., Sczesnak A. (2017). IgA-coated *E. coli* enriched in Crohn’s disease spondyloarthritis promote TH17-dependent inflammation. Sci. Transl. Med..

[85] David L.A., Maurice C.F., Carmody R.N., Gootenberg D.B., Button J.E., Wolfe B.E., Ling A.V., Devlin A.S., Varma Y., Fischbach M.A. (2014). Diet rapidly and reproducibly alters the human gut microbiome. Nature.

[86] Brown K., DeCoffe D., Molcan E., Gibson D.L. (2012). Diet-induced dysbiosis of the intestinal microbiota and the effects on immunity and disease. Nutrients.

[87] Ebringer A., Wilson C. (1996). The use of a low starch diet in the treatment of patients suffering from ankylosing spondylitis. Clin. Rheumatol..

[88] Cao G., Wang Q., Huang W., Tong J., Ye D., He Y., Liu Z., Tang X., Cheng H., Wen Q. (2017). Long-term consumption of caffeine-free high sucrose cola beverages aggravates the pathogenesis of EAE in mice. Cell Discov..

[89] Di Luccia B., Crescenzo R., Mazzoli A., Cigliano L., Venditti P., Walser J.-C., Widmer A., Baccigalupi L., Ricca E., Iossa S. (2015). Rescue of fructose-induced metabolic syndrome by antibiotics or faecal transplantation in a rat model of obesity. PLoS ONE.

[90] Macfarlane T.V., Abbood H.M., Pathan E., Gordon K., Hinz J., Macfarlane G.J. (2018). Relationship between diet and ankylosing spondylitis: A systematic review. Eur. J. Rheumatol..

[91] Andoh A., Bamba T., Sasaki M. (1999). Physiological and anti-inflammatory roles of dietary fiber and butyrate in intestinal functions. J. Parenter. Enter. Nutr..

[92] Looijer-van Langen M.A., Dieleman L.A. (2009). Prebiotics in chronic intestinal inflammation. Inflamm. Bowel. Dis..

[93] Christl S.U., Eisner H.D., Dusel G., Kasper H., Scheppach W. (1996). Antagonistic effects of sulfide and butyrate on proliferation of colonic mucosa: A potential role for these agents in the pathogenesis of ulcerative colitis. Dig. Dis. Sci..

[94] Tan J., McKenzie C., Vuillermin P.J., Goverse G., Vinuesa C.G., Mebius R.E., Macia L., Mackay C.R. (2016). Dietary fiber and bacterial scfa enhance oral tolerance and protect against food allergy through diverse cellular pathways. Cell Rep..

[95] Zhernakova A., Kurilshikov A., Bonder M.J., Tigchelaar E.F., Schirmer M., Vatanen T., Mujagic Z., Vila A.V., Falony G., Vieira-Silva S. (2016). Population-based metagenomics analysis reveals markers for gut microbiome composition and diversity. Science.

[96] Willett W.C., Sacks F., Trichopoulou A., Drescher G., Ferro-Luzzi A., Helsing E., Trichopoulos D. (1995). Mediterranean diet pyramid: A cultural model for healthy eating. Am. J. Clin. Nutr..

[97] Sureda A., Del Mar Bibiloni M., Julibert A., Bouzas C., Argelich E., Llompart I., Pons A., Tur J.A. (2018). Adherence to the Mediterranean diet and inflammatory markers. Nutrients.

[98] Dinu M., Pagliai G., Casini A., Sofi F. (2018). Mediterranean Diet and multiple health outcomes: An umbrella review of meta-analyses of observational studies and randomised trials. Eur. J. Clin. Nutr..

[99] Poggioli R., Hirani K., Jogani V.G., Ricordi C. (2023). Modulation of inflammation and immunity by omega-3 fatty acids: A possible role for prevention and to halt disease progression in autoimmune, viral, and age-related disorders. Eur. Rev. Med. Pharmacol. Sci..

[100] Niu Q., Wei W., Huang Z., Zhang J., Yang B., Wang L. (2019). Association between food allergy and ankylosing spondylitis: An observational study. Medicine.

[101] Bowness P. (2015). HLA-B27. Annu. Rev. Immunol..

[102] Taurog J.D., Richardson J.A., Croft J.T., Simmons W.A., Zhou M., Fernández-Sueiro J.L., Balish E., Hammer R.E. (1994). The germfree state prevents development of gut and joint inflammatory disease in HLA-B27 transgenic rats. J. Exp. Med..

[103] Ciccia F., Dussias N.K., Gandolfo S., Rizzello F., Gionchetti P. (2024). The effect of anti-TNF drugs on the intestinal microbiota in patients with spondyloarthritis, rheumatoid arthritis, and inflammatory bowel diseases. Rheumatol. Immunol. Res..

